# From Few to Many: New World Founded by Surprisingly Small Population

**DOI:** 10.1371/journal.pbio.0030227

**Published:** 2005-05-24

About 14,000 years ago—a few hundred thousand years after our putative modern forebears spread out from Africa—descendants of archaic humans crossed the Bering land bridge from Siberia to North America. Several lines of evidence support this model, but that's where the consensus ends. The details remain hotly debated, focusing mostly on which Asian population migrated, when they did it, and whether they did it more than once.

Part of the challenge in reconstructing this history stems from the dynamic nature of human populations—which experience unpredictable changes in size, composition, density, and mating patterns—and the difficulty in interpreting genetic history. To get a better picture of the range of possible scenarios, scientists are using new statistical approaches that require computer simulations. Jody Hey now extends this approach in a novel method for the study of the origins of New World populations. Along with DNA analysis and computer simulations, Hey adds a new twist to an old model to reveal how the sizes of the first New World populations have changed since they were founded. His results fall in line with archeological, genetic, and linguistic evidence, pointing to a relatively recent colonization of the Americas. But they go a step further by showing that the New World was colonized by a small population with an effective size of only about 70 individuals. (The effective size refers to the number of individuals likely to contribute genes to the next generation.)

Hey's approach addresses shortcomings in population genetic studies that rely on just one gene and that assume that population sizes have been constant over time. Studying levels of DNA sequence variation at a single genomic region, or loci, can offer insight into the history of that gene, but the stochastic nature of gene evolution means that different genes have different histories. And as simplified versions of a very complex reality, population genetics models, such as the “isolation with migration” (IM) model, that aim to capture the population dynamics during the early stages of divergence or speciation have necessary limitations. The widely used IM model, for example, assumes that a founding population splits into two descendant populations that may interbreed, and incorporates a large number of parameters. But until now the IM model has required the assumption that all of the populations were constant in size, and therefore it has not been useful for assessing how descendant populations arose or changed in size.[Fig pbio-0030227-g001]

**Figure pbio-0030227-g001:**
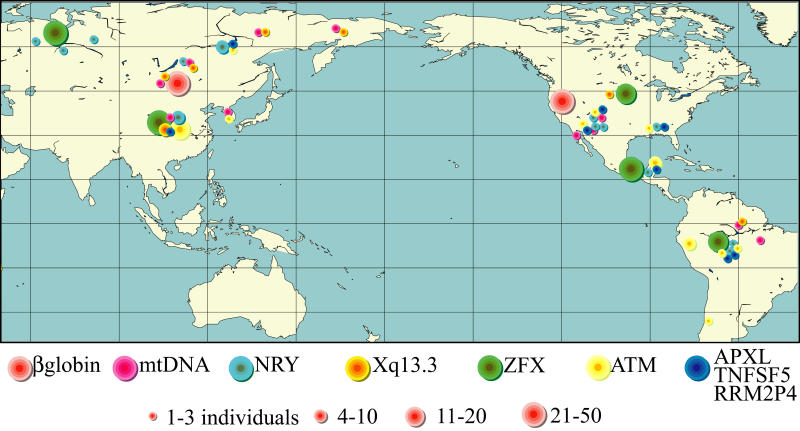
Data from nine different regions in the human genome chart the journey of the first immigration to the New World

Hey analyzed DNA sequences from nine loci, so that the population genetic history could be found despite the variation among genes. He also added an additional parameter to the standard IM model to incorporate changes in the size of the ancestral population and of each founder population through time. The genetic data included DNA sequences from Asian and American Indian individuals. Hey varied the parameters in his model, which included founding population size, changes in population size, time of population formation (and splitting), and gene exchange between the populations, to work out the demographic scenario that best fit the available genetic data.

His analysis suggests that only about 70 individuals left their ancestral Asian population, estimated at about 9,000 individuals, to reach America 7,000 to 14,000 years ago. Archeological evidence places the earliest American inhabitants in the New World at around 14,000 years ago. Though Hey's estimates are more recent, they also indicate a high probability at this time period. Hey did not include genetic data from Eskimo-Aleut and Na Déne speakers, so the number of migrations was not addressed. But with this new approach, researchers will be able to explore this and many other questions to fill in the details of the first American immigration.

